# Native extracellular matrix: a new scaffolding platform for repair of damaged muscle

**DOI:** 10.3389/fphys.2014.00218

**Published:** 2014-06-16

**Authors:** Laura Teodori, Alessandra Costa, Rosa Marzio, Barbara Perniconi, Dario Coletti, Sergio Adamo, Bhuvanesh Gupta, Attila Tarnok

**Affiliations:** ^1^UTAPRAD-DIM, ENEA FrascatiRome, Italy; ^2^Fondazione San RaffaeleCeglie Messapica, Italy; ^3^Department of Surgery, McGowan Institute, University of Pittsburgh Medical CenterPittsburgh, PA, USA; ^4^UMR 8256 CNRS Biology of Adaptation and Aging, University Pierre et Marie Curie Paris 06Paris, France; ^5^Section of Histology and Medical Embryology, Department of Anatomical, Histological, Forensic and Orthopaedic Sciences, Sapienza University of RomeRome, Italy; ^6^Department of Textile Technology, Indian Institute of TechnologyNew Delhi, India; ^7^Department of Pediatric Cardiology, Heart Centre Leipzig, and Translational Centre for Regenerative Medicine, University of LeipzigLeipzig, Germany

**Keywords:** biomaterials, extracellular matrix, tissue engineering, regenerative medicine, skeletal muscle, scaffold, decellularization

## Abstract

Effective clinical treatments for volumetric muscle loss resulting from traumatic injury or resection of a large amount of muscle mass are not available to date. Tissue engineering may represent an alternative treatment approach. Decellularization of tissues and whole organs is a recently introduced platform technology for creating scaffolding materials for tissue engineering and regenerative medicine. The muscle stem cell niche is composed of a three-dimensional architecture of fibrous proteins, proteoglycans, and glycosaminoglycans, synthesized by the resident cells that form an intricate extracellular matrix (ECM) network in equilibrium with the surrounding cells and growth factors. A consistent body of evidence indicates that ECM proteins regulate stem cell differentiation and renewal and are highly relevant to tissue engineering applications. The ECM also provides a supportive medium for blood or lymphatic vessels and for nerves. Thus, the ECM is the nature's ideal biological scaffold material. ECM-based bioscaffolds can be recellularized to create potentially functional constructs as a regenerative medicine strategy for organ replacement or tissue repopulation. This article reviews current strategies for the repair of damaged muscle using bioscaffolds obtained from animal ECM by decellularization of small intestinal submucosa (SIS), urinary bladder mucosa (UB), and skeletal muscle, and proposes some innovative approaches for the application of such strategies in the clinical setting.

## Introduction

Tissue engineering aims to mimic neo-organogenesis to produce *ex-vivo* living tissue (Carosio et al., 2013). Initial clinical experiences with bioengineered tissues have been reported in skin, cartilage, vascular grafts, bones, and several other specialized internal tissues, such as liver and kidney (Olson et al., 2011). However, owing to its intrinsic complexity, skeletal muscle remains a challenge for *in vitro* tissue engineering. Most engineered muscle structures have been obtained by employing an artificial scaffold, such as matrigel (Lü et al., 2009, 2012), or native or modified collagen (van Wachem et al., 1996; Okano and Matsuda, 1997, 1998). Decellularization of tissues and whole organs is a recently introduced platform technology for creating scaffolding materials composed of an extracellular matrix (ECM) for skeletal muscle tissue engineering. The resulting bioscaffolds (i.e., scaffold of biological origin) can then be recellularized to create potentially functional constructs as a regenerative medicine strategy for organ replacement or tissue repopulation. Indeed, the ECM represents the secreted product of the resident cells of each tissue or organ. It includes both functional and structural molecules arranged in a unique three-dimensional ultrastructure that supports the phenotype and the function of the resident cells (Reing et al., 2009, 2010). Appropriate tissue decellularization preserves not only the ECM integrity, bioactivity and spatial structure, but also the vascular, lymphatic and nervous network (Badylak et al., 2012). Moreover, a native ECM scaffold obtained by means of decellularization is also biodegradable, thereby responding to another important requirement of an ideal biomaterial for tissue engineering. Thus, a tissue-derived ECM is the ideal bioscaffold, and all the components that are retained during its preparation are likely to contribute to the success of the ECM upon implantation. Indeed, the ECM is not merely a static entity that supports the tissues, but plays a critical role in cell signaling and tissue homeostasis, provides molecules for cell-matrix interactions (such as laminin and fibronectin), maintains the appropriate physico-chemical properties, and represents a fundamental structure for mechano-transduction signals (Chiquet, 1999; Badylak et al., 2012). The ECM helps to structure niches spatially and modulate the concentration of adhesive and signaling molecules locally (Kim et al., 2011). A niche is considered as a subset of tissue, cells and extracellular substrates (matrix and soluble factors) that support stem cells and control their self-renewal *in vivo* (Escobedo-Lucea et al., 2012). In this regard, recent studies provide strong evidence that the niche is composed of both soluble factors and ECM macromolecules that direct cell fate (Brown and Badylak, 2014). Thus, the niche represents a specialized local microenvironment that contributes to the establishment and maintenance of the stem cell phenotype and stem cell differentiation (Jones and Wagers, 2008). Indeed, the use of ECM-derived scaffolds in tissue engineering is strictly dependent on its niche properties in stem cell recruitment and differentiation. When implanted *in vivo*, an ECM-derived scaffold elicits an immune response from the host (Keane and Badylak, 2014), which in turn is responsible for one of the key events of tissue regeneration/remodeling: ECM-derived scaffold degradation. Some authors have demonstrated that ECM degradation releases both growth factors such as basic fibroblast growth factor (bFGF), vascular endothelial growth factor (VEGF), insulin-like growth factor, hepatocyte growth factor (Crapo et al., 2012; Choi et al., 2013; Hoganson et al., 2010), and cryptic peptides (Agrawal et al., 2011a,b; Ricard-Blum and Ballut, 2011) which activate cell surface receptors (Davis, 2010) and are required for cell viability, motility, and differentiation (Voytik-Harbin et al., 1997; Hodde et al., 2001, 2005; Badylak et al., 2009). They all may subsequently induce the events that lead to tissue regeneration. In particular, these factors are hypothesized to polarize the macrophage phenotype toward an M2 anti-inflammatory phenotype (Turner and Badylak, 2013), rather than toward an M1 pro-inflammatory phenotype, and to recruit different stem or progenitors cells that may give rise to new tissue formation (Agrawal et al., 2011a,b), vasculature and innervation (Agrawal et al., 2009; Sicari et al., 2012; Turner et al., 2012). Indeed, it has recently been demonstrated that native ECM scaffolds from skeletal muscle elicit M2 macrophage polarization during the host inflammatory response (Valentin et al., 2009; Turner and Badylak, 2013). M2 macrophages play a key role in the resolution of inflammation as well as in the activation of satellite cells during skeletal muscle regeneration (Kharraz et al., 2013). In skeletal muscle tissue engineering, the use of an ECM-derived scaffold *in vivo* has been shown to recruit CD133^+^ cells (Turner et al., 2012), recently identified as progenitors of a myogenic cell population, as well as Sca1^+^/PW1^+^ cells identified as muscle interstitial stem cells, named PICs (Perniconi et al., 2011), Sox2+, and Sca1+, Lin- cells (Agrawal et al., 2011a,b). The suggestion that ECM degradation products directly affect macrophage polarization is also supported by evidence indicating that chemically cross-linked ECM scaffolds, which are not biodegradable. The fact that the ECM of each specific tissue has a specific biochemical composition and 3D structure that influence the host response through the release of suitable GFs (Hoganson et al., 2010; Agrawal et al., 2011a,b; Crapo et al., 2012; Choi et al., 2013) and of specific cryptic peptide (which are retained after decellularization and are released during ECM degradation), implies that the tissue specific ECM may elicit the growth and differentiation of those tissue specific cells and has some advantages over ECM from non-homologous tissues.

The maintenance of 3D architecture has a significant relevance to the regeneration of complex organs and tissues. In particular, skeletal muscle functionality is strictly dependent on the correct alignment of myofibers (Boontheekul et al., 2007; Klumpp et al., 2010). A native ECM scaffold from skeletal muscle tissue presumably preserves the correct architecture of the native ECM surrounding each myofiber. On the basis of these considerations, a novel approach to tissue engineering of skeletal muscle, which involves the use of three-dimensional bioscaffolds made of an allogeneic or xenogeneic ECM derived from skeletal muscle tissue, has been proposed. The large scaffolds required for this approach may be of cadaveric origin and may to some extent be stored. However, the harvesting of muscle tissue from the same patient may be considered for minor defects or for plastic surgery. Owing to the scarcity of studies on skeletal muscle-derived ECM, the present review investigates the reconstruction of skeletal muscle tissue based on both skeletal muscle and non-skeletal muscle (SIS and UB) ECM decellularized tissue. The state of the art regarding studies on ECM derived from decellularized tissues for skeletal muscle tissue regeneration/remodeling worldwide, their current applications in clinics and future perspective are also discussed. A review of cartilage- and bone-derived ECM bioscaffold production and use, as opposed to skeletal muscle-derived ECM, has been recently published (Cheng et al., 2014).

## Decellularization of tissues for ECM-based scaffold production

Decellularization is the first step of a strategy that attempts to obtain a biologically engineered construct that resembles the native tissue or organ as closely as possible (Badylak et al., 2012). This strategy is particularly suitable for complex tissues or organs that require the maintenance of spatial architecture for translational applications.

Processing methods play a critical role in determining the type of host response (Valentin et al., 2009; Faulk et al., 2014a,b). The challenge faced by each decellularization method is to completely remove the cellular component and DNA content (less than 50ng of dsDNA per 1 mg dry weight of ECM scaffold) while preserving the ECM biochemical features, architecture, ultrastructure, and porosity (Crapo et al., 2011). Decellularization protocols are based on physico-chemical agents, enzymes, detergent solutions, or a combination of these (Crapo et al., 2011). Physical reactions, such as freeze-thaw cycles, and mechanical forces, such as hydrostatic pressure, are sufficient to induce cell lysis. Chemical agents include: per-acetic and acetic acid, which remove nucleic acids but affect collagen; bases (such as NaOH), which destroy growth factors; hypertonic or hypotonic solutions, which lyse cells through osmotic shock (Crapo et al., 2011). Commonly used detergents include Triton X-100 and CHAPS for thick tissues, or low percentages of SDS (sodium dodecyl sulfate) for whole organ decellularization. These detergents solubilize the cell membrane and disrupt DNA (Meyer et al., 2006; Jones and Wagers, 2008; Giusti et al., 2009; Petersen et al., 2010; Reing et al., 2010; Crapo et al., 2011), though they have negative effects on protein and ultrastructure. Chelating agents, such as EDTA (ethylenediaminetetraacetic acid) and EGTA (ethylene glycol tetraacetic acid), help to dissociate cells from the ECM, while serine protease inhibitors, such as PMSF (phenylmethylsulfonyl fluoride), aprotinin, and leupeptin, prevent ECM damage (Crapo et al., 2011). Several types of enzymes, such as nuclease, trypsin, and collagenase, can be used to specifically and quantitatively perform some of the afore-mentioned tasks. A balance must be achieved between chemical and physical treatments during the decellularization process (Macchiarini et al., 2008; Ott et al., 2008; Escobedo-Lucea et al., 2012). The most effective agent for decellularization of a specific tissue or organ depends on many factors, including the tissue size, cellularity, density, lipid content, and thickness. For thin tissue laminae, such as the urinary bladder, intestine, pericardium, and amnion, decellularization techniques include freezing and thawing, mechanical removal of muscle or submucosa, and brief treatment with detergents or acid (Crapo et al., 2011). For thick tissue laminae, such as dermis, a more extensive biochemical exposure and a longer incubation time are required. Adipose tissue, brain, and pancreas typically require the addition of lipid solvents (Crapo et al., 2011). For whole organ decellularization, the action of detergent and biological agents is enhanced by antegrade or retrograde perfusion, as demonstrated for the heart (Ott et al., 2008; Wainwright et al., 2010), lung, liver, and other organs (Petersen et al., 2010; Price et al., 2010; Shupe et al., 2010; Bonvillain et al., 2013; Tsuchiya et al., 2014), or even by agitation in the decellularization solution, as is done for the majority of tissues (Crapo et al., 2011; Perniconi et al., 2011). As far as skeletal muscle tissue decellularization is concerned two of the most recent methods are very different. Some authors described a method requiring several days for tibialis anterior murine muscle (TA), based on treatment with latrunculin B that disrupts actin, osmotic shock for cell lysis, and ionic salt solution for depolymerizing myosin. The resulting bioscaffold is completely decellularized, but preserves the amount of collagen and glycosaminoglycans (GAGs) required, the overall architecture of the native ECM, and the mechanical integrity (Gillies et al., 2011). By applying the same protocol, Fishman et al. (2013) also achieved the decellularization of rabbit cricoarytenoid dorsalis muscle (CAD). The second method (Perniconi et al., 2011) is based on a 1% SDS detergent solution and requires only 48 h to obtain a murine TA scaffold. This method resulted in the decellularization of skeletal muscle, while preserving the biochemical features (such as collagen, laminin, and fibronectin) and 3D-architecture of the native ECM. Moreover, when implanted to replace the whole host homologous muscle *in vivo*, this bioscaffold represented a pro-myogenic environment (Perniconi et al., 2011). Previous studies that have addressed skeletal muscle tissue engineering using a native-ECM approach were based on long and complex decellularization processes. One such example is the decellularization of rat abdominal muscles achieved by means of a protocol based on osmotic shock, 4% sodium deoxycholate and DNase-I, which required more than 3 days (Conconi et al., 2005). The resulting scaffold supported myoblast growth and differentiation *in vitro*. When the decellularized muscle seeded with myoblasts was implanted between the *obliquus externus abdominis* and the *obliquus internus abdominis*, neovascularization and the formation of new myofibers occurred within 2 months (Conconi et al., 2005). Merritt et al. decellularized a rat gastrocnemius (GAS) by means of a protocol based on osmotic shock and detergent solutions that required several days (Merritt et al., 2010b). The resulting decellularized GAS was used as a patch to repair a muscle defect: the graft did not elicit an immune response and was capable of supporting the growth of myofibers (between 28 and 42 days after transplantation) and blood vessels (between 7 and 28 days after transplantation). More recently, Fishman et al. (2013) decellularized rat TA by cycles of freeze-thaw and distilled water for 72 h, followed by enzymatic treatment (trypsin) and Triton X-100 for a further 4 days. The acellular native ECM was transplanted to repair a volumetric muscle injury. Two weeks after transplantation, the graft displayed extensive infiltration of CD68 positive cells, few regenerating myofibers in the areas surrounding the injured muscle and vascularization (Wu et al., 2012). To sum up, the afore-mentioned studies demonstrate (i) that native ECM-derived scaffold from various tissues can be obtained by means of different protocols that may require several days (Gilbert et al., 2006), and (ii) that this scaffold supports growth and survival of myogenic cells both *in vitro* and *in vivo*, and that it represents a pro-myogenic environment.

The importance of decellularization methods and their effect upon the resulting ECM structure and composition is currently highly relevant, as is demonstrated by the fact that at least two other reviews, besides ours, are being published in 2014. Since the advantages and drawbacks of several decellularization methods have been systematically reviewed (Badylak, 2014; Brown and Badylak, 2014; Faulk et al., 2014a,b), we recommend that they be referred to for details on these issues. We believe that each of these studies unveils important information on the native-ECM derived scaffold used to reconstruct skeletal muscle tissue: on its ability to support cell viability and growth *in vitro*, to provide a pro-myogenic environment *in vivo*, to elicit an immune response polarized toward an M2 macrophage phenotype and to preserve its molecular and structural features, as well as on the time and mode of decellularization and possible storage conditions. However, none provides details leading to the full reconstruction of a whole muscle. An overview of the literature on the topic is shown in Table 1.

**Table 1:** **Overview of the literature on ECM-based bioscaffolds**.

**Who, When**	**Where**	**Source**	**Target**
Agrawal et al., 2009	University of Pittsburgh, Pennsylvania, USA	Porcine urinary bladder matrix	Canine oesophagus; rat abdominal muscular wall
Alberti and Xu, 2013	Tufts University, Medford, USA	Bovine achilles tendon	Potential role for nerve guidance conduits and blood vessel tissue engineering
Borschel et al., 2004	University of Michigan, Ann Arbor, USA	Murine skeletal muscles	C2C12 culture *in vitro*
Chen and Walters, 2013	United States Army Institute of Surgical Research, Extremity Trauma and Regenerative Medicine Research Program, San Antonio, TX, USA, and Wake Forest Institute for Regenerative Medicine, Winston-Salem, NC, USA	Rat muscle-derived extracellular matrix	Reconstruction of VML in rat latissimus dorsi
Carmignac and Durbeej, 2012	Lund University, Lund, Sweden	ECM-cell membrane-cytoskeleton interactions	Treatment of muscle disorders
Conconi et al., 2005	University of Padova, Italy	Rat skeletal muscle seeded with myoblasts	Muscle fibers in syngeneic host
Corona et al., 2013	US Army Institute of Surgical research, Fort Sam, Houston, USA	Rat skeletal muscle	Enhanced mechanical stability in VML
Dai and Xu, 2011	Tufts University, Medford, USA	Adult cow tendons	Future application in other natural materials, e.g., muscles
Gillies et al., 2011	University of California San Diego, La Jolla, USA	Murine skeletal muscles	C2C12 culture *in vitro*
Mase et al., 2010	Institute of Surgical Research, Houston, USA	Porcine intestinal submucosa	Human right thigh medialis muscle
Merritt et al., 2010a	University of Texas, Austin, USA	Rat muscle-derived ECM seeded with bone-marrow-derived mesenchymal stem cells	Rat lateral gastrocnemius skeletal myofibers
Milner and Cameron, 2013	University of Illinois, Urbana, USA	Skeletal muscle	Amphibian limbs
Perniconi et al., 2011	Sapienza University of Rome, Rome, Italy	Murine skeletal muscles	Muscle fibers in syngeneic host
Sicari et al., 2012	University of Pittsburgh, Pennsylvania, USA	Porcine intestinal submucosa	VML in murine quadriceps
Stern et al., 2009	Wake Forest University School of Medicine, Winston-Salem, USA	Hamster skeletal muscles	Coating for C2C12 colture *in vitro*
Turner et al., 2010	University of Pittsburgh, Pennsylvania, USA	Xenogeneic ECM	Dog distal gastrocnemius musculotendinous junction
Turner et al., 2012	University of Pittsburgh, Pennsylvania, USA	Dog small intestinal submucosa	Dog quadriceps skeletal muscle
Valentin et al., 2010	University of Pittsburgh, Pennsylvania, USA	Carbodiimide-crosslinked porcine SIS; autologous tissue; polypropylene mesh	Rodent abdominal wall
Vindigni et al., 2004	University of Padua, Italy	Rat rectus abdominis seeded with satellite cells	Reconstruction of homologous muscle
Wolf et al., 2012	University of Pittsburgh, Pennsylvania, USA	Dog skeletal muscle; porcine small intestine submucosa	Rat abdominal muscular wall
Wu et al., 2012	University of Texas, San Antonio, Texas, USA	Rat tibialis anterior muscle	Rat tibialis anterior muscle

*List of works dealing with the production, characterization, and use of ECM-derived bioscaffolds obtained by means of tissue/organ decellularization and proposed for tissue engineering applications*.

## Characterization of ECM bioscaffolds

### From xenogenic matrixes to skeletal muscle

The biomechanical and biochemical properties of ECM scaffolds derived from porcine small intestinal submucosa (SIS) and porcine urinary bladder mucosa (UB) have been well characterized (Derwin et al., 2006; Freytes et al., 2008a,b). Porcine SIS ranges in thickness from 0.05 to 0.22 mm and has a variable porous microstructure, though it is always sufficient for oxygen diffusion to sustain cell proliferation and viability and is biodegradable in 3 months. It is primarily composed of type I collagen fibers, but also contains a minor amount of elastin and collagen types III, IV, and VI. Multi-domain glycoproteins such as fibronectin and laminin, which mediate cell adhesion to the extracellular matrix, have also been identified in SIS. Moreover, SIS contains glycosaminoglycans and proteoglycans that provide cell attachment and growth factor binding sites, sequesters matrix-degrading enzymes and enhances cellular infiltration into injured tissue. It has also been documented that SIS releases growth factors, including FGF-2, TGF-β 1, and VEGF (Voytik-Harbin et al., 1997; Badylak et al., 1998; Mase et al., 2010). In a body wall repair, a canine model SIS has been shown to elicit the formation of new ECM associated with skeletal muscle and adipose tissue regeneration (Badylak et al., 2002). Thus, SIS is an ideal remodelable biomaterial for muscle tissue engineering, thanks to its size, thin membranous configuration, relative uniformity, and availability. SIS has so far been used to repair inguinal hernia and large abdominal wall defects, the urinary tract, achilles tendon, musculotendinous tissues and dura mater, and as vascular graft material and for dermal wounds (Prevel et al., 1995; Cobb et al., 1996, 1999; Dejardin et al., 1999, 2001; Kropp, 1999; Shalhav et al., 1999; Portis et al., 2000; Badylak et al., 2002).

ECM scaffold materials derived from porcine UB have been extensively studied recently. Two ECM bioscaffolds can be derived from adjacent layers of porcine UB: urinary bladder matrix and urinary bladder sub mucosa, derived from tunica mucosa including the basement membrane and from tunica sub mucosa, respectively. The advances made in ECM scaffold technology have led to the development, manufacturing, and commercialization of a naturally-occurring porcine urinary bladder matrix (Lecheminant and Field, 2012; Kruper et al., 2013; Rommer et al., 2013). It has been demonstrated that it allows the retention of multiple growth factors including VEGF, transforming growth factor beta (TGFβ), platelet derived growth factor (PDGF), bone morphogenetic protein 4 (BMP4), and basic fibroblast growth factor (BFGF), which contribute to regeneration and healing. Recent studies have demonstrated the antimicrobial properties of UB-ECM to both *S. aureus* and *E. coli* (Brennan et al., 2006) as well as a pre-dominance of macrophage phenotype 2 in the early post-operative period, which has been shown to reduce scar formation and enhance healthy functional tissue regeneration with increased chemotactic activity (Turner and Badylak, 2013).

### From skeletal muscle to skeletal muscle

In spite of the extensive characterization of SIS and UB, as yet very little information has been provided on ECM derived from skeletal muscle tissue. The need for a bioscaffold that preserves the native 3D-architecture of the ECM is particularly evident for skeletal muscle tissue engineering applications, in which the correct parallel alignment of myofibers and the maintenance of mechanical properties are essential for tissue functionality. Indeed, the only extensive report demonstrating that decellularized skeletal muscle preserved its biochemical features, such as collagen, laminin, and fibronectin, as well as the 3D-architecture of the native tissue, is the one by Perniconi et al. (2011). Their study provides evidence indicating that such a muscle ECM-derived scaffold provides the correct support for myofiber development and the appropriate architecture for muscle fiber formation. In particular, when this bioscaffold was orthotopically grafted in syngeneic animals, the activation of myogenic cells and the formation of new myofibers in areas within the bioscaffold was observed. However, 4 weeks after transplantation, the grafts had almost completely degraded, probably owing to digestion by neutrophils and macrophages. In this regard, immunosuppressors such as Cyclosporin A have been reported to enhance the myogenic process and delay graft digestion (Perniconi et al., 2011). The decellularized bioscaffold, together with the removal of the host muscle, is likely to have elicited an inflammatory response dominated by neutrophils and macrophages. The inflammatory process peaked 2 weeks after transplantation and then declined. This process was, however, accompanied by the presence of stem cells expressing PW1, a global stemness marker described by Sassoon and co-workers (Besson et al., 2011) and by the formation of new myofibers within the bioscaffold from 1 to 3 weeks after transplantation. In the 4th week, the graft started undergoing biodegradation (Perniconi et al., 2011). This study also demonstrated that the decellularized scaffold can be stored for several weeks in sterile conditions at either +4°C in PBS or +36°C in DMEM in culture conditions, though laminin preservation in the latter case was worse. The possibility of storing the acellular scaffold is highly relevant to the use of such devices in future clinical applications. Other recent skeletal muscle acellular scaffold preparations (described in the decellularization section) from TA and CAD have been shown to retain the overall architecture of native ECM. In particular, Gillies et al. (2011) demonstrated that TA-derived acellular scaffolds not only maintained their collagen and glycosaminoglycan (GAG) levels, the overall architecture of the native ECM, their mechanical integrity, but also supported murine myogenic cells C2C12 survival *in vitro*. By contrast, decellularized CAD implanted in the rat abdominal wall modulated the immune response, polarizing macrophages toward the M2 phenotype and inducing T-cell hypo-responsiveness (Fishman et al., 2013).

## State of the art of ECM bioscaffold grafting

Although the use of the decellularized ECM for skeletal muscle tissue engineering is a recent innovation, the results of studies that have been published to date on this topic are promising. Indeed, the majority of data have demonstrated that the ECM can support muscle and blood vessel regeneration, though recovery of function is not complete (Merritt et al., 2010b).

Animal models, consisting mainly of rats, mice, rabbits, and dogs subjected to experimentally induced skeletal muscle injury, have been used as recipients of xenogeneic ECM scaffold grafts to test surgical treatment for volumetric muscle loss. To our knowledge, such treatment has been applied to humans a very limited number of times. One such case was described in a case report by Mase et al., who used porcine SIS to restore multiple injuries in the quadriceps muscle in a young soldier (Mase et al., 2010). Implantation of the SIS supported by intensive physical therapy improved the pick torque, total work, and average power of the grafted limb 16 weeks post-operatively (Mase et al., 2010). A very recent study demonstrated the remodeling characteristics of xenogeneic porcine SIS ECM bioscaffolds when used to surgically treat volumetric muscle loss in five male patients (Badylak et al., 2012; Sicari et al., 2014). That study showed that ECM bioscaffold implantation was associated with perivascular stem cell mobilization and accumulation within the site of injury and *de novo* formation of skeletal muscle cells. Overall, the studies in the literature demonstrate that ECM bioscaffold implantation recruits stem cells within the bioscaffold and promotes differentiation and *de novo* formation of skeletal muscle cells. Although the mechanism of action underlying stem and progenitor cell recruitment is not yet fully understood, it might be ascribed to extracellular matrix chemotactic cryptic peptide (Agrawal et al., 2011a,b; Ribeiro et al., 2012) and the host's immune response, which induces the macrophage 2 phenotype (Brown et al., 2012; Turner and Badylak, 2013). This is in agreement with our observation that macrophages largely account for the inflammatory invasion of muscle ECM-derived bioscaffolds, and are consequently likely to play a major role in muscle graft integration (Perniconi et al., 2011). The ability of bioscaffolds to alter the macrophage phenotype response, coupled with the release of latent growth factors and chemotactic degradation products, means these materials lend themselves to being used as scaffolds to promote skeletal muscle reconstruction following trauma and volumetric loss (Mase et al., 2010; Turner and Badylak, 2013). The clinical results of these applications may vary for a wide range of reasons that include not only the characteristics of the source tissue, methods, and efficacy of tissue decellularization, and methods of processing/manufacturing, but also the response of the host to these implanted biological scaffold materials, which may hamper the success of the implant. Although several studies on the remodeling characteristics of ECM scaffolds are currently in progress, to the best of our knowledge a systematic temporal evaluation of the structural and functional muscle remodeling following the implantation of acellular scaffolds is still lacking in the literature.

## Problems and challenges

The use of ECM biologic scaffolds for skeletal muscle tissue reconstruction is attracting a high degree of interest in regenerative medicine. Recent advances have opened new perspectives for the replacement of skeletal muscle tissue in clinical applications, such as traumatic injury and pathological conditions. However, these highly promising applications in the field of regenerative medicine require a greater understanding of the biochemical, cellular, and mechanical mechanisms that stimulate the constructive remodeling response. Although ECM bioscaffolds implanted in an injury site have been shown to promote the migration and proliferation of progenitor cells, the triggers that cause these cells to differentiate into site appropriate tissue are still unknown. Moreover, a better characterization of ECM bioscaffolds derived from different tissues may help to select ECM bioscaffolds that are most suited to constructive remodeling in different sites. Improved methods of decellularization and ECM bioscaffold preparation may improve the retention and release of growth factors, and consequently increase the success of implantation. Last but not least, improved quality control should be aimed at the optimization of ECM scaffold decellularization and implantation so as to enhance the likelihood of survival and reduce that of rejection (Koch et al., 2012). For example, image cytometry technologies of tissues (also referred to as tissue cytometry Heindl et al., 2013) could easily be adapted to quantify the remaining cells or cell fragments on scaffolds after staining with appropriate fluorescent markers for DNA, RNA, organelles, specific cells, etc. By adopting suitable approaches, image cytometry may also be adopted for acellular or hypocellurar tissues (Fueldner et al., 2012). Moreover, like all cytometry techniques, tissue cytometry may be standardized to optimize reproducibility (Mittag and Tarnok, 2009), and since tissue cytometry can be multiplexed by using different colors for different targets (Gerner et al., 2012), minimal specimen amounts are needed. Non-invasive label free detection methods may also be applied (Nallala et al., 2013), implying that this technique may prove useful for pre-implantation quality assessment. Last, despite progresses made in the research and clinical application of skeletal muscle tissue engineering in recent decades, further efforts aimed at whole muscle engineering are required. Indeed, the use of generic ECM scaffolds for a wide range of applications are unlikely to be sufficient for complex organs, and ECM derived from homologous organs may be required to support site specific differentiation of appropriate cells. Investigations on whole organ scaffolds are needed to shed further light on this issue in the future.

### Conflict of interest statement

The authors declare that the research was conducted in the absence of any commercial or financial relationships that could be construed as a potential conflict of interest.
